# Supplementary material for: Effective Reduction in Nuclear DNA Contamination Allows Sensitive Mitochondrial DNA Methylation Determination by LC-MS/MS
Source: Int J Mol Sci. 2025 Sep 11;26(18):8864. doi: 10.3390/ijms26188864 (PMC12469381; doi:10.3390/ijms26188864)
Supplement: Supplementary file 1 [file ijms-26-08864-s001.zip › ijms-3810053-supplementary.pdf]

Supplementary

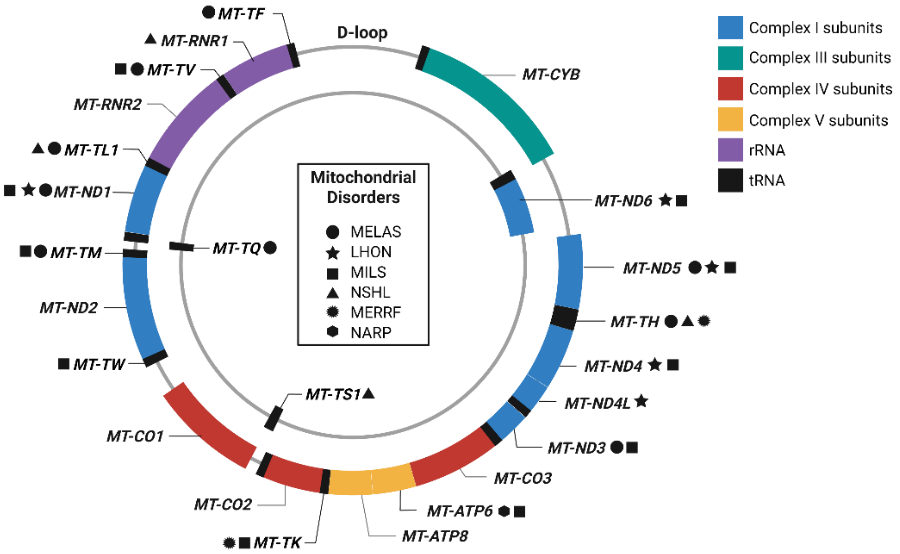

Supplementary Figure S1. Examples of mitochondrial disorders stemming from mtDNA mutations. These disorders are characterized by metabolic impairment leading to neurological manifestations. MELAS: Mitochondrial encephalomyopathy lactic acidosis and stroke like episodes; LHON: Leber hereditary optic neuropathy; MILS: maternally inherited Leigh's syndrome; NSHL: Non-syndromic hearing loss; MERRF: Myoclonic epilepsy with ragged red fibers; NARP: neurogenic weakness with ataxia and retinitis pigmentosa. Figure created with BioRender.com.

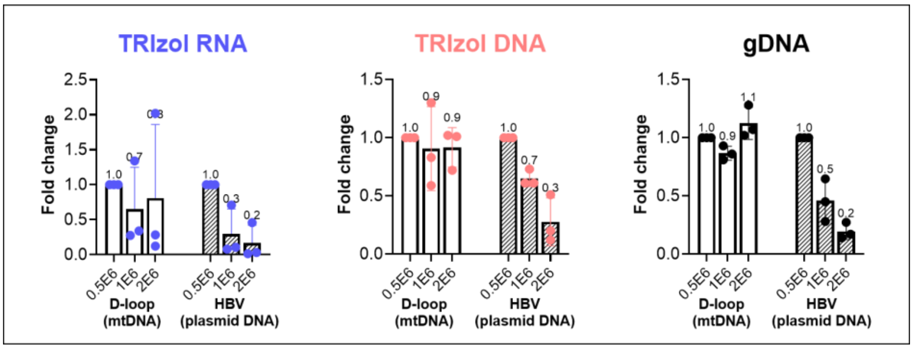

Supplementary Figure S2. variable mtDNA and HBV 1.3-mer plasmid DNA content in TRIzol RNA and TRIzol DNA phase measured by qPCR. 50ng plasmid DNA was added to twofold increasing numbers of HepG2 cells. Fold change represents the relative difference between $2x10^6$ and 0.5 $x10^6$ cells, as well as $1x10^6$ and 0.5 $x10^6$ cells. MtDNA relative content was normalized with *GAPDH* DNA in gDNA and TRIzol DNA samples, and normalized with *GAPDH* cDNA in TRIzol RNA samples. DNase treatment was performed prior to cDNA synthesis according to the DNase I, RNase-free (Thermo Scientific) manufacturer protocol. The cDNA synthesis was performed with the Revertaid cDNA synthesis kit (Thermo Scientific). (n=3)

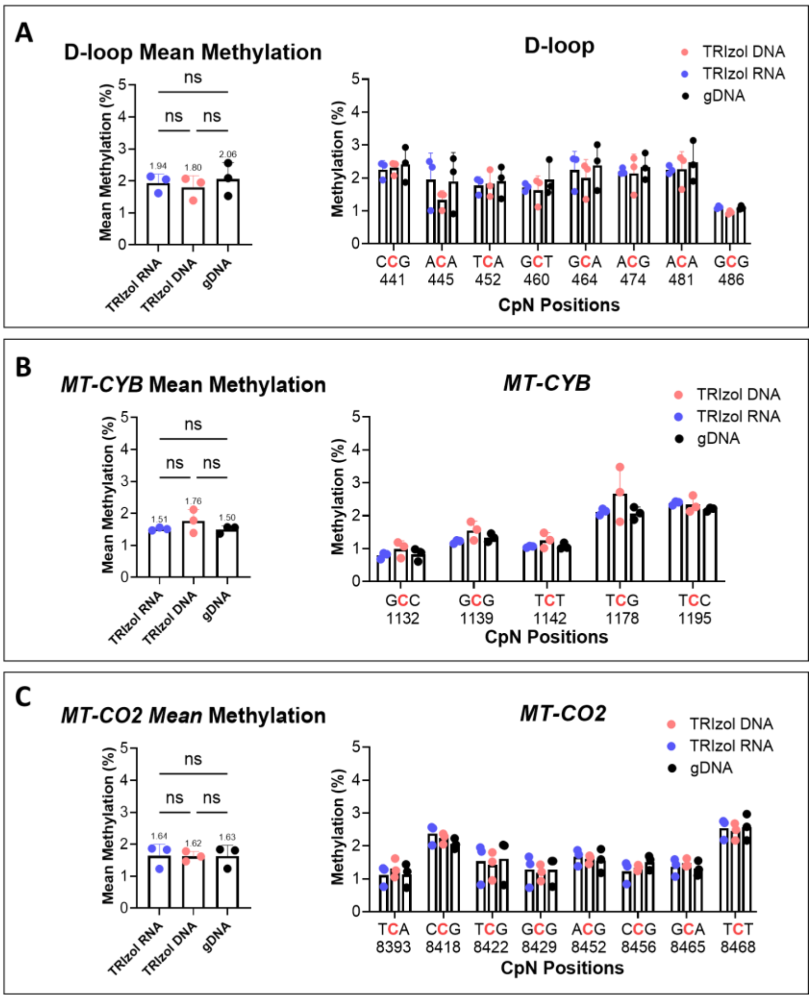

Supplementary Figure S3. MCF7 mtDNA methylation measured by pyrosequencing. Methylation percentages of mtDNA regions **A)** D-loop, **B)** *MT-CYB,* and **C)** *MT-CO2*, are shown for TRIzol RNA, TRIzol DNA and gDNA isolates. The H-strand was targeted. Mean methylation was calculated as the mean of all CpN positions in the targeted regions. Statistical significance was determined using paired t test ($p < 0.05$), ns: no significant difference (n=3).

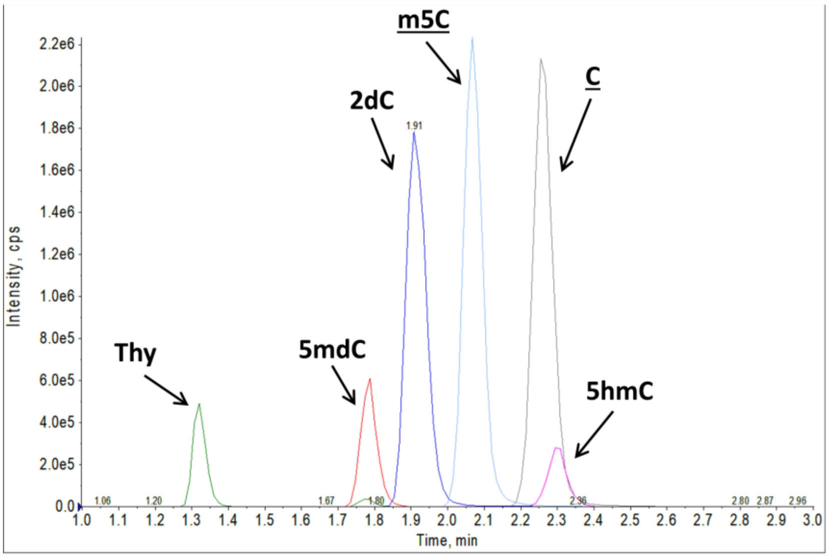

Supplementary Figure S4. Representative chromatogram of the nucleosides. Intensity is shown on the y axis, and retention time on the x axis. Each chromatogram is normalized to the same signal intensity. All nucleosides are marked with its abbreviation and two of the RNA nucleosides are underlined. Thy (thymidine), 5mdC (5-methyl-2'-deoxycytidine), 2dC (2'-deoxycytidine), m5C (5-methylcytidine), C (cytidine) and 5hmC (5-(hydroxy) methyl-2'-deoxycytidine).

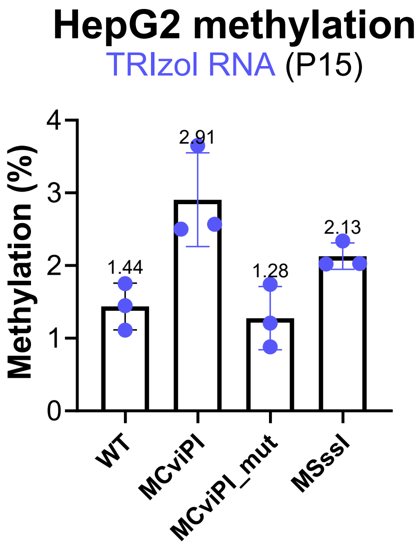

Supplementary Figure S5. DNA methylation in HepG2 transgenic cell lines. DNA was isolated by TRIzol RNA for cell lines from the same passage (P15). DNA methylation was measured by LC-MS/MS. MCviPI represents HepG2 cell transduced with MCviPI methylation enzyme to induce GpC methylation, MCviPI_mut represents the MCviPI mutant cell line, MSssI represents HepG2 cell transduced with MSssI methylation enzyme to induce CpG methylation (n=3).

Supplementary Table S1. qPCR primers sequences.

Primer	Sequence
MT-CYB	Fw: AATTCTCCGATCCGTCCCTA<br>Rv: GGAGGATGGGGATTATTGCT
D-loop	Fw: TATTTTCCCCTCCCACTCCCA<br>Rv: TTCGGGGTATGGGGTTAGCA
GAPDH DNA	Fw: CCCTTCATACCCTCACGTATTC<br>Rv: CCATTCTGTCTTCCACTCACTC
GAPDH cDNA	Fw: CCACATCGCTCAGACACCAT<br>Rv: GCGCCCAATACGACCAAAT
ACTB DNA	Fw: TGAGTGGCCCGCTACCTCTT<br>Rv: CGGCAGAAGAGAGAACCAGTGA
HBV	Fw: CGTCTGTGCCTTCTCATCTG<br>Rv: GTCCTCTTGTGTAAGACCTTGG
mt-Co2 (mouse)	Fw: ACCTGGTGAACTACGACTGCT<br>Rv: TCCTAGGGAGGGGACTGCTC
Gapdh (mouse)	Fw: TGGCAAAGTGGAGATTGTTGCC<br>Rv: AAGATGGTGATGGGCTTCCCG

Supplementary Table S2. Pyrosequencing primers sequences. Universal biotin label primer is marked bold.

Region	Target Location	Primer sequence
D-loop	441-565	Fw: GGTTGATTGTTGTATTTGTTTGTAAGT<br>Rv: **GGACACCGCTGATCGTTTA**CACCATTAACACCCAAAACTAAAATTCTA<br>Seq: TTTATGTATTATAGGTGGTTAAG
MT-CYB	1132 - 1195	Fw: GAGGTTTGGTGAGAATAGTGTT<br>Rv: **GGGACACCGCTGATCGTTTA**CTTTACCTTTCACTTCATCTTACC<br>Seq: AAGGAGAGAAGGAAGA
MT-CO2	8388-8522	Fw: ATTGTGGTTTGTTTTATAGATTTTAGAG<br>Rv: **GGGACACCGCTGATCGTTTA**TTACACTCATAAACTATCCCCACAT<br>Seq: TGTTTTATAGATTTTAGAGTATTGA

Supplementary Table S3. Pyrosequencing targeted mtDNA sequence. Targeted cytosines are marked red bold.

Region	Target Location	mtDNA sequence and targeted cytosines
D-loop	441-565 (H-strand 5'-3')	**C**GTA**C**AATATT**C**ATGGTGG**C**TGG**C**AGTAATGTA**C**GAAATA**C**ATAG**C** GGTTGTTGATGGGTGAGTCAATACTTGGGTGGTACCCAAATCTGCT TCCCCATGAAAGAACAGAGAATAGTTTAAATTA
MT-CYB	1132 - 1195 (H-strand 5'-3')	**C**CGAGGG**C**GT**C**TTTGATTGTGTAGTAAGGGTGGAAGGTGATTTTAT **C**GGAATGGGAGGTGATT**C**
MT-CO2	8388-8522 (H-strand 5'-3')	GATTT**C**AGAGCATTGACCGTAGTATACCCC**C**GGT**C**GTGTAG**C**GGTG AAAGTGGTTTGGTTTAGA**C**GTC**C**GGGAATTG**C**AT**C**TGTTTTTAAGCC TAATGTGGGGACAGCTCATGAGTGCAAGACGTCTTGTGATGT

Supplementary Table S4. LC-MS/MS transitions for all analytes.

Analytes	Precursor ion (m/z)	Product ion (m/z)	Declustering potential (eV)	Collision energy (eV)
2'Deoxycytidine (quantifier)	228.1	112	20	17.5
2'Deoxycytidine (qualifier)	228.1	95	20	49
2'-Deoxycytidine-15N3 (quantifier)	231.05	115	8	17.5
2'-Deoxycytidine-15N3 (qualifier)	231.05	97	8	49
5-Methyl-2'-deoxycytidine (quantifier)	242.1	126	10	18
5-Methyl-2'-deoxycytidine (qualifier)	242.1	109	10	50
5-Methyl-2'-deoxycytidine -d3 (quantifier)	245.1	129	10	18
5-Methyl-2'-deoxycytidine -d3 (qualifier)	245.1	112	10	50
5-Hydroxymethyl-2'-deoxycytidine (quantifier)	258.1	142.1	10	15
5-Hydroxymethyl-2'-deoxycytidine (qualifier)	258.1	124.1	10	31
5-Hydroxymethyl-2'-deoxycytidine -d3 (quantifier)	261.1	145.1	10	15
5-Hydroxymethyl-2'-deoxycytidine -d3 (qualifier)	261.1	127.1	10	31
Thymidine	243.1	127.1	30	17
Thymidine-13C10_15N2	255.1	134.1	30	17
Cytidine	244	112	25	25
Me-Cytidine	258	126	25	25

Supplementary Table S5. LC-MS/MS inter-assay and intra-assay imprecision.

		Inter-assay (n=6)			Intra-assay (n=10)		
		**Mean**	**SD**	**CV%**	**Mean**	**SD**	**CV%**
**2'-Deoxycytidine**	**low**	11.02	0.46	4.16	11	0.70	6.10
	**high**	41.9	3.9	9.20	49	2.1	4.28
**5-Methyl-2'-deoxycytidine**	**low**	0.10	0.021	22.23	0.11	0.01	5.98
	**high**	0.38	0.029	7.75	0.36	0.02	5.47
**5-Hydroxymethyl-2'-deoxycytidine**	**low**	0.00	0.00	< LOQ	0.00	0.00	< LOQ
	**high**	0.00	0.00	< LOQ	0.00	0.00	< LOQ
**Thymidine**	**low**	9.82	0.84	8.57	11	0.77	6.99
	**high**	37	2.7	7.29	47	3.8	8.17
**Cytidine**	**low**	1.6	0.30	18.58	2.0	0.01	0.28
	**high**	1.6	0.30	18.40	2.0	0.01	0.49
**5-Methylcytidine**	**low**	0.00	0.00	< LOQ	0.36	0.00	0.32
	**high**	0.00	0.00	< LOQ	0.36	0.00	0.28

Red marked values are the imprecision of the detected analytes > 15%, due to its low concentration in the HBV plasmid (5-Methyl-2'-deoxycytidine) or the absence of its isotope labelled internal standards (5-Methylcytidine).

Supplementary Table S6. LOD and LOQ of LC-MS/MS.

**Analytes**	**LOD** (ng/mL)	**LOQ** (ng/mL)
2'-Deoxycytidine	0.021	0.064
5-Methyl-2'-deoxycytidine	0.003	0.008
5-Hydroxymethyl-2'-deoxycytidine	0.009	0.029
Thymidine	0.1	0.304
Cytidine	0.013	0.038
5-Methylcytidine	0.011	0.034

LOD: limit of detection, calculated by $3.3x\ \sigma / S$. LOQ: limit of quantification, calculated by $10x\ \sigma / S$, where S is the slope and σ is the standard error of the Y-intercept.
